# Glucose-Stimulated Mucus Secretion by Goblet Cells Mitigates Intestinal Barrier Dysfunction in a Rat Model of Mesenteric Ischemia/Reperfusion Injury

**DOI:** 10.1016/j.cdnut.2024.104431

**Published:** 2024-07-26

**Authors:** Ting-You Guo, Wei-Ting Kuo, Yi-Syuan Tsai, Linda Chia-Hui Yu, Ching-Ying Huang

**Affiliations:** 1 Department of Food Science and Biotechnology, National Chung Hsing University, Taichung, Taiwan; 2 Graduate Institute of Oral Biology, College of Medicine, National Taiwan University, Taipei, Taiwan; 3 Department of Dentistry, College of Medicine, National Taiwan University, Taipei, Taiwan; 4 Department of Dentistry, National Taiwan University Hospital, Taipei, Taiwan; 5 Graduate Institute of Physiology, National Taiwan University College of Medicine, Taipei, Taiwan

**Keywords:** glucose, goblet cell, ischemia reperfusion, mucus, bacterial translocation, gut barrier function

## Abstract

**Background:**

Superior mesenteric ischemia/reperfusion (I/R) causes barrier dysfunction and facilitates bacterial translocation (BT) in the small intestine, which can even lead to systemic sepsis. Our previous research showed that luminal administration of glucose and its anaerobic glycolytic metabolites exerted cytoprotective effects on epithelial cells and ameliorated I/R-induced BT in the liver and spleen. Notably, the reduction of BT occurs over the whole intestinal tract, not only restricted in the ligated glucose-containing loop.

**Objectives:**

In this study, we hypothesized that local jejunal glucose-contacting might confer on the remote intestinal epithelium regeneration potential, fortify their barrier function and goblet cell secretory activity.

**Methods:**

Two 10-cm jejunal segments were isolated in Wistar rats. One segment was ligatured at both ends and infused with Krebs buffer containing 0- or 50-mM glucose (local loop), whereas the adjacent segment was left unaltered and not exposed to glucose (remote loop). The rats then underwent either a sham operation or I/R challenge by occlusion of the superior mesenteric artery for 20 min, followed by reperfusion for 1 h.

**Results:**

Enteral addition of glucose in the local jejunum loop alleviated ischemia-induced barrier defects, histopathological scores, cell death, and mucosal inflammation (myeloperoxidase and inflammatory cytokine production) in the remote jejunum. After ischemia, goblet cells in the remote jejunum showed cavitation of mucin granules and low MUC2 expression. Local addition of glucose enhanced MUC2 synthesis and stimulated a jet-like mucus secretion in the remote jejunum, which was accompanied by the restoration of crypt activity.

**Conclusions:**

Our results showed local enteral glucose effectively mitigates I/R-induced barrier dysfunction, suggesting that local glucose-stimulated mucus secretion by remote goblet cells may serve to mitigate mucosal inflammation and BT. We provide a more precise barrier protection role of enteral glucose upon I/R challenge, presenting new opportunities for future therapeutic potential.

## Introduction

During intestinal injury, loss of mucus and barrier disruption can lead to increased bacterial adhesion, invasion, and mucosal inflammation, increasing risk of intestinal bacterial translocation (BT) into visceral organs and possibly leading to sepsis. Production of mucus is mainly performed by goblet cells and is essential for the maintenance of the structural and functional integrity of the intestinal mucosa. For this reason, goblet cells are considered to be crucial components of the protective intestinal barrier and key guardians against a wide range of diseases. The unique rheological properties of mucus can be attributed to gel-forming glycoproteins known as mucins, which are continuously produced by goblet cells located primarily within the intestinal epithelium [1,2]. Various intestinal disorders, such as ischemia/reperfusion (I/R) [3], ulcerative colitis [[4], [5], [6], [7]], and necrotizing enterocolitis [8], typically involve goblet cell dysfunction or other causes of reduced mucus secretion. The decreased mucus can further disrupt the ecological balance of intestinal bacteria and cause dysbiosis.

In clinical practice, intestinal I/R injury stems from both predictable and unpredictable causes. Predictable events include planned medical procedures such as intestinal transplants and surgeries for abdominal aortic aneurysms or chronic mesenteric ischemia. Unpredictable causes may include acute mesenteric ischemia, resuscitation from cardiac events, surgical complications, or neonatal issues. Although the unpredictable scenarios generally lack level 1 evidence to guide treatment and management, these causes are often associated with high mortality. For predictable causes, early intervention has been shown to greatly mitigate risk of severe complications caused by intestinal barrier damage due to mesenteric artery ischemia [9]. However, the role of mucus secreted by goblet cells in preventing damage caused by I/R remains largely unknown.

Our prior studies showed that enteral administration of glucose within a jejunal loop of rats offers protection against superior mensenteric artery (SMA) I/R-induced barrier defects. The observed effects of glucose treatment include the prevention of epithelial cell death (apoptosis and necroptosis), reduced mucosal inflammation, and restoration of cryptic proliferation. When the glucose concentration in the intestinal lumen is under 50 mM, the sodium-dependent glucose transporter 1 (SGLT1) is vital for apical glucose uptake into intestinal cells, whereas glucose transporter 2 (GLUT2) on the basolateral membrane enables the passive transfer of glucose to the bloodstream [10,11]. The nutrient supply to small intestine cells is distinctive, because the tissue can derive nutrients from both hematologic and dietary sources. At times when the blood supply cannot provide sufficient nutrients, enteral nutrition can offer an alternative pathway for cells to receive nutrients. As such, providing adequate nutrition directly to the intestinal lumen can support the maintenance of intestinal barrier integrity, preventing breakdown of the barrier and subsequent bacterial influx under ischemia conditions. Notably, in addition to the cytoprotective effects of glucose during SMA I/R, we also discovered that local enteral glucose administration in the ischemic jejunal loop inhibits BT over the whole intestinal tract, not only restricted in the ligated glucose-containing loop [12,13]. However, it remains unclear how enteral glucose administration in the local jejunal loop would prevent global BT in the intestines.

Goblet cell is the secretory lineage of differentiated intestinal epithelial cells. The mucus secreted by goblet cells predominantly consists of the gel-forming mucin protein MUC2, which is extensively glycosylated. The mucus layer in the small intestine is a single, unattached layer with a thickness of ∼150–300 µm, whereas the colon has a dual-layer system comprised of a dense, attached inner layer and a less dense, unattached outer layer [14,15]. During the maturation of mucins in goblet cells, the proteins are oligomerized and glycosylated within the endoplasmic reticulum (ER) and Golgi apparatus, which confers the characteristic viscosity and protective properties [2]. Mucus serves as both an immunomodulator and a physical barrier in the intestines, separating luminal contents from the epithelial surface [[16], [17], [18]]. In the small intestine, mucin forms a net-like structure that traps bacteria and sequesters the bacterial cells away from the epithelium, except at the villus tip; this sequestration is enhanced by antibacterial peptides secreted from crypt Paneth cells [14]. This arrangement limits bacterial diffusion and maintains a buffer between bacteria and host cells, and it also ensures a bacteria-free zone in the crypt area that enhances the effectiveness of antibacterial peptides [19,20]. Studies have shown that I/R in the colon is generally less damaging than I/R in the small intestine [[21], [22], [23]]. This difference may be due to the dual-layer mucus system of the colon, which can rapidly counteract ischemic effects through increased secretory activity of goblet cells [3,23]. However, it remains largely unknown how goblet cells and mucus secretion are regulated after I/R in the small intestine, and it also remains unclear what impacts these responses have on barrier function.

In this study, we tested whether local administration of glucose in the jejunum could strengthen the barrier function of distant intestinal epithelium and enhance its regenerative capabilities. To understand how local glucose administration inhibits BT in the rest of the intestine, we utilized a dual intestinal loop model in rats. In this model, glucose is administered to one loop, whereas the other loop is not exposed to glucose. This approach allowed us to examine the impacts of glucose on local and remote mucus-mediated barrier function after I/R.

## Methods

### Rat models of intestinal ischemia/reperfusion

All animal protocols used in the present study were approved and monitored by the Institutional Animal Care and Use Committee at the National Chung Hsing University (IACUC No. 108-084, 110-126). Wistar rats weighing between 250 and 300 g were fasted overnight and provided with unrestricted access to water. The rats were then subjected to sham surgery or mesenteric I/R challenge, following previously described procedures [12,13,24,25]. After anesthesia, the absence of the withdrawal reflex was confirmed, and the rat underwent a midline laparotomy. In rats receiving I/R, the SMA was occluded with an atraumatic microvascular clamp for 20 min and then released for 60 min. Control rats receiving sham surgery were subjected to simulated manipulation of the SMA and killed at the same time as the I/R group.

### Experimental design

**FIGURE 1:**
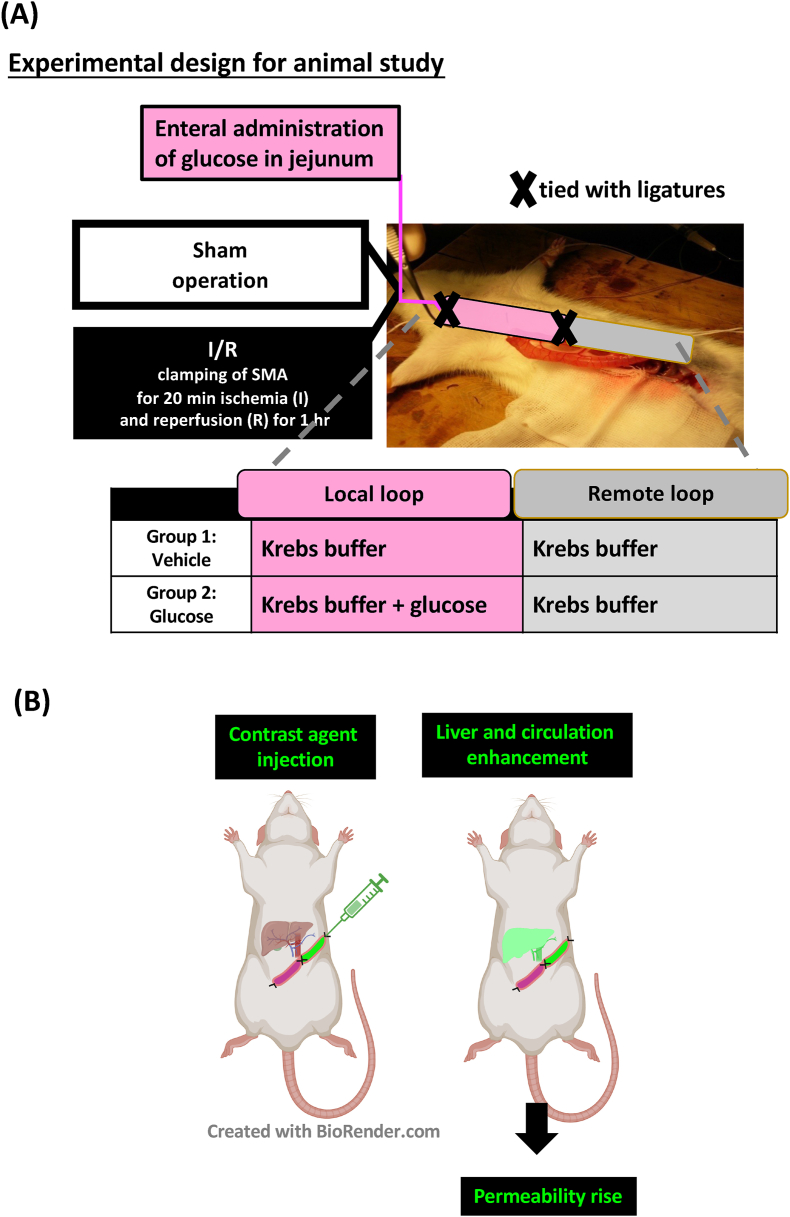
Experimental design of the dual small intestinal loop model. (A) A 10-cm loop of the jejunum (beginning 10 cm distal to the ligament of Treitz) was ligatured and infused with a Krebs buffer containing either 0 or 50 mM glucose (local jejunum). The adjacent intestinal loop was left unaltered and designated as the remote loop. (B) Schema of the experimental protocols for MRI-based intestinal permeability assay. The local jejunum loop was infused with a Krebs buffer containing either 0 or 50 mM glucose. The ligated remote intestinal loop was instilled with the contrast agent after SMA I/R or mock manipulation of the artery. Plasma sample was collected postreperfusion and then quantified using MRI as indices of intestinal permeability. I/R, ischemia/reperfusion; SMA, superior mensenteric artery.

The experimental protocols were carried out under aseptic conditions. After anesthetization, all rats were subjected to midline laparotomy. A dual small intestinal loop procedure was performed in rats, wherein a 10-cm loop of the local jejunum was ligatured and infused with a Krebs buffer, containing either 0 or 50 mM glucose. The treated loop was referred to as the local jejunum (Figure 1A). The loop of the jejunum adjacent to this was left unaltered and was referred to as the remote jejunum. The local jejunal loop was created by thread ligature at both ends, beginning 10 cm distal to the ligament of Treitz in each animal. Care was taken not to occlude or puncture mesenteric vessels during the ligation. Intubation at one end of the local jejunal loop was performed using a 1-mL syringe with a PE-10 catheter, and 1 mL Krebs buffer with glucose (Sigma) was carefully injected into the lumen. The Krebs buffer was prepared as follows: 115 mM NaCl, 8 mM KCl, 1.25 mM CaCl_2_, 1.2 mM MgCl_2_, 2.0 mM KHPO_4_, 25 mM NaHCO_3_, at pH 7.33–7.37. Animals were then subjected to sham operation or I/R challenge as described above. At the end of the surgical procedures, the tissue from local and remote small intestines were collected for experimental analysis.

### Histopathological scoring and goblet cell quantification

Intestinal tissues were fixed in 4% paraformaldehyde and processed for paraffin embedding. The embedded tissues were used for histological examination and goblet cell quantification. After sectioning, the jejunal tissues were stained with H&E or Alcian Blue and Periodic acid-Schiff (PAS; Abcam). Images were captured under light microscopy. The degree of intestinal injury was graded under light microscopy by 2 independent evaluators (T-Y.G. and C-Y.H.) who were blinded to the treatment, as previously described [13]. Briefly, intestinal injury was scored from 0 to 5 according to the following criteria: grade 0, normal mucosal villous structure; grade 1, presence of subepithelial space at villous tips; grade 2, scattered epithelial denudation on villous tips; grade 3, denuded tips with exposed lamina propria and villous blunting; grade 4, epithelial shedding from both the apex and mid-region of the villi associated with shortened and widened villous structure; grade 5, complete destruction of villi and disintegration of lamina propria with ulceration. For quantification of PAS-positive cells in gut mucosa, 5 fields of view from each animal were quantified; a total of 5 rats per group were evaluated. The results were calculated as number of goblet cells/villus.

### Magnetic resonance imaging–based gut permeability assay

For the real-time evaluation of intestinal permeability in vivo, the contrast agent gadodiamide (0.25 M Gd; Omniscan; GE Healthcare) was introduced into the lumen of the tied-off jejunal section immediately after the arterial clamp was removed (Figure 1B). The plasma signal intensity from this agent was then quantified using MRI, following previously described methods [12,13,24].

### Immunohistochemical and immunofluorescence staining

The preparation of intestinal tissue sections for immunostaining was carried out according to previously established protocols [12,13]. In summary, tissue sections were first blocked with 2% normal goat serum and then incubated with primary antibodies against proliferating cell nuclear antigen (PCNA) at a dilution of 1:100 (Lifespan Biosciences), Ki67 at a dilution of 1:100 (Abcam), Zonula occludens (ZO)-1 at a dilution of 1:500 (clone R40.76, DHSB), occludin at a dilution of 1:100 (clone 6B8A3, DHSB), cleaved caspase-3 at a dilution of 1:100 (clone 5A1E, Cell Signaling), or isotype control antibodies. Following rinses in phosphate-buffered saline (PBS), sections designated for PCNA detection underwent further incubation with horseradish peroxidase–conjugated anti-rabbit immunohistochemistry detection reagent (Cell Signaling Technology), and a signal was generated using a 3,3'-diaminobenzidine peroxidase substrate kit; hematoxylin was used for counterstaining. For Ki67, ZO-1, Occludin, and cleaved caspase-3 labeling, sections were treated with Alexa 488-conjugated secondary antibodies at a dilution of 1:1000 (Invitrogen); cell nuclei were labeled with Hoechst stain. For mucus staining, Carnoy fixative-exposed samples were stained with a mixture of 20 μg/mL Wheat Germ Agglutinin (WGA) (Invitrogen) and 1 μg/mL Hoechst stain (Thermo Fisher Scientific) for 15 min at room temperature, followed by 3 5-min washes with PBS. Finally, the sections were covered with an aqueous mounting solution and examined under fluorescence microscopy (Carl Zeiss). To quantify cell proliferation in intestinal tissues, the numbers of Ki67-positive cells per crypt were calculated from 5 well-oriented crypts in longitudinal view from each animal; a total of 6 rats per group were analyzed. Zen software (Zeiss) was used for quantitative analysis of immunostaining. Annotation of epithelial villi and crypt areas was performed manually.

### Western blot of MUC2

Jejunal mucosa samples were homogenized in ice-cold radioimmunoprecipitation assay (RIPA) buffer with complete protease inhibitors, followed by sonication and centrifugation. Protein concentrations in the supernatant were normalized to 5 mg/mL and mixed with an equal volume of ×2 electrophoresis sample buffer, which included 2% SDS, 100 mM DTT, and 62.5 mM Tris/HCl at pH 6.8. The mixture was then heated at 95°C for 5 min in a heat block and stored at −20°C for subsequent immunoblotting.

For protein analysis, SDS-PAGE was performed to separate the proteins, which were then transferred to membranes. The membranes were blocked using 5% nonfat milk in TBS and incubated overnight at 4°C with an anti-MUC2 antibody (1:1000; Abcam). To ensure equal protein loading, a monoclonal mouse anti–beta-actin antibody (1:10000; Sigma) was used. After washing with TBS containing 0.1% Tween20, the membranes were incubated with horseradish peroxidase–conjugated secondary antibodies (goat anti-rabbit or anti-mouse IgG, 1:1000; Cell Signaling) to detect the bound antibodies. Protein bands were visualized and quantified through photoimage analysis.

### Myeloperoxidase (MPO) activity assay

Intestinal tissues were processed by homogenization and sonication in a solution containing 10 tissue volumes of 50 mM potassium phosphate buffer (PPB) at pH 6.0, with 0.5% hexadecyltrimethylammonium bromide (HTAB, Sigma). Following centrifugation, the supernatants were further diluted in PPB mixed with 0.167 mg/mL O-dianisidine dihydrochloride (Sigma), and a low concentration of H_2_O_2_. The concentration of the enzyme was ascertained by recording the absorbance at 460 nm at 30-s intervals for 5 min. One unit of MPO activity was defined as the amount of enzyme that catalyzes the decomposition of 1 mmol of H_2_O_2_ per minute. The MPO activity in intestinal tissue was quantified as units per milligram of tissue.

### ELISA for tumor necrosis factor (TNF)-α and macrophage inflammatory protein (MIP)-1α

The mucosal layers scraped from jejunum tissues were homogenized and sonicated in PBS, followed by centrifugation. The protein content of the resulting supernatant was determined. TNF-α and MIP-1α concentrations in the mucosal samples were assessed using ELISA kits from PeproTech, according to the guidelines provided by the manufacturer. For cytokine quantification, microplates were prepared by overnight coating with specific capture antibodies, and then blocked for 1 h in PBS with 1% bovine serum albumin. After washing the wells, samples and standards were added to the plates and were allowed to incubate for 2 h. This was followed by a 2-h incubation with a biotinylated detection antibody specific to the antigen of interest. After a series of washes, an avidin–horseradish peroxidase (HRP) conjugate was applied for 30 min. Color development was achieved by adding 2,2′-azino-bis-(3-ethylbenzothiazoline-6-sulfonic) acid (ABTS) liquid substrate, and the absorbance was measured at 405 nm (correction wavelength, 650 nm). The levels of cytokines in the jejunal mucosa were reported in picograms per milligram of protein.

### BT assay

Following the I/R procedure, the rats' spleens and livers were surgically removed and weighed using sterile instruments. The tissues were then homogenized with a tissue homogenizer (PRO Scientific Inc.) and diluted to a concentration of 0.1 g/mL in PBS. Each homogenate sample (200 μL per plate) was spread onto fresh blood agar plates (Scientific Biotech Corp.) and incubated at 37°C overnight to detect live bacteria in these organs. The number of bacterial CFUs was determined and expressed as CFU per gram of tissue.

### Statistical analysis

All values are expressed as mean ± SEM. Data were compared by one-way analysis of variance followed by a Student–Newman–Keul test or analysis of variance followed by Fisher’s least significant difference test using GraphPad Prism (GraphPad Software). For the bacterial CFU, the value was expressed as median, and data were compared by nonparametric Mann–Whitney *U* test. Statistical significance was set at *P* < 0.05.

## Results

Our previous studies showed that administering glucose to a jejunal loop can reduce I/R-induced epithelial cell death (including apoptosis and necroptosis), decrease intestinal damage, and enhance barrier function in the intestines of rats subjected to I/R injury [12,13]. Moreover, we found that administering glucose in a 10-cm segment of the jejunum can reduce the overall translocation of intestinal bacteria to the liver and spleen [13]. In this study, we examined 2 adjacent 10-cm segments of the jejunum (local and remote) and tested whether administering glucose to the local jejunum can reduce I/R-induced barrier defects in the remote intestine (Figure 1A).

### Local enteral glucose protects against I/R-induced loss of intestinal tight junctions and increased permeability in the remote loop

**FIGURE 2:**
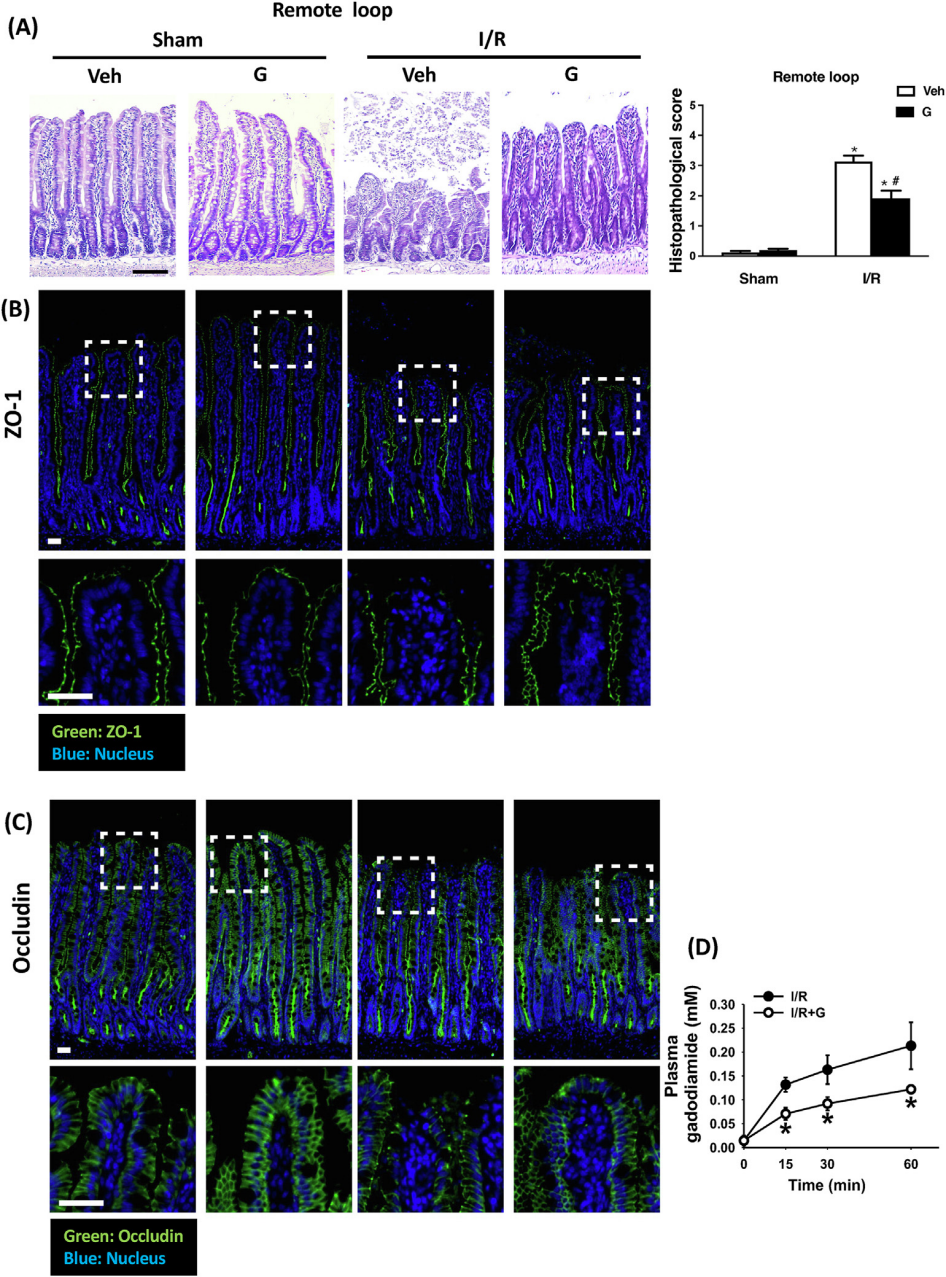
Local enteral glucose treatment attenuates mesenteric ischemia/reperfusion (I/R)-induced barrier defects in the remote small intestinal loop. Intestinal tissue sections from the remote small intestinal loop were processed for H&E staining. (A) Representative histological images are shown. I/R-induced mucosal destruction was significantly reduced in animals treated with enteral glucose (indicated as G) (Scale bar: 100 μm). Photomicrographs of small intestine tissues stained for the tight junctional protein (B) ZO-1 (green) and (C) occludin (green). Nuclei were stained with Hoechst (blue). Arrows indicate sites of apical surface disruption in ZO-1- and occludin-stained remote intestine after I/R. The disruption of tight junction proteins was prevented by local enteral glucose. (Scale bar: 20 μm for B and C). (D) MRI-based gut permeability assay performed by enteral exposure to MRI contrast agent Gadodiamide (MW 574 Da) in the remote small intestinal loop. Permeability changes were assessed postreperfusion. Local enteral glucose prevented increased intestinal permeability in I/R-exposed rats. (*N* = 6–8/group, ∗*P* < 0.05 vs. Sham + Veh, ^#^*P* < 0.05 vs. IR + Veh.). G, glucose; H&E, hematoxylin and eosin; I/R, ischemia/reperfusion; Veh, vehicle; ZO, zonula occludens.

Consistent with our previous findings, inducing mesenteric ischemia for 20 min followed by reperfusion for 60 min resulted in villous destruction and denudation, as well as epithelial cell sloughing in both the local and remote loops of the intestine (Figure 2A, Supplemental Figure 1A). The mucosal damage was accompanied by a disappearance of tight junctional proteins (i.e., ZO-1 and occluding) normally expressed in the villous tip (Figure 2B, C, Supplemental Figure 1B, C). Administering glucose to the local jejunum alleviated I/R-induced mucosal injury in both the local and remote intestinal loops (Figure 2A, Supplemental Figure 1A), as both loops exhibited more intact structures and were covered by complete epithelial layers over the villi. The glucose-associated preservation of ZO-1 and occludin expression was comparable in the remote and local loops (Figure 2B, C, Supplemental Figure 1B, C). Utilizing an MRI-based permeability assay (Figure 1B), we also found that enteral administration of glucose in the local jejunum could suppress the increase of intestinal permeability in the remote intestine, suggesting that local glucose administration enhances barrier function in both the local and remote intestine (Figure 2D).

### Local enteral glucose protects against I/R-induced caspase-3 cleavage in the villus tip and preserves crypt activity in the remote loop

**FIGURE 3:**
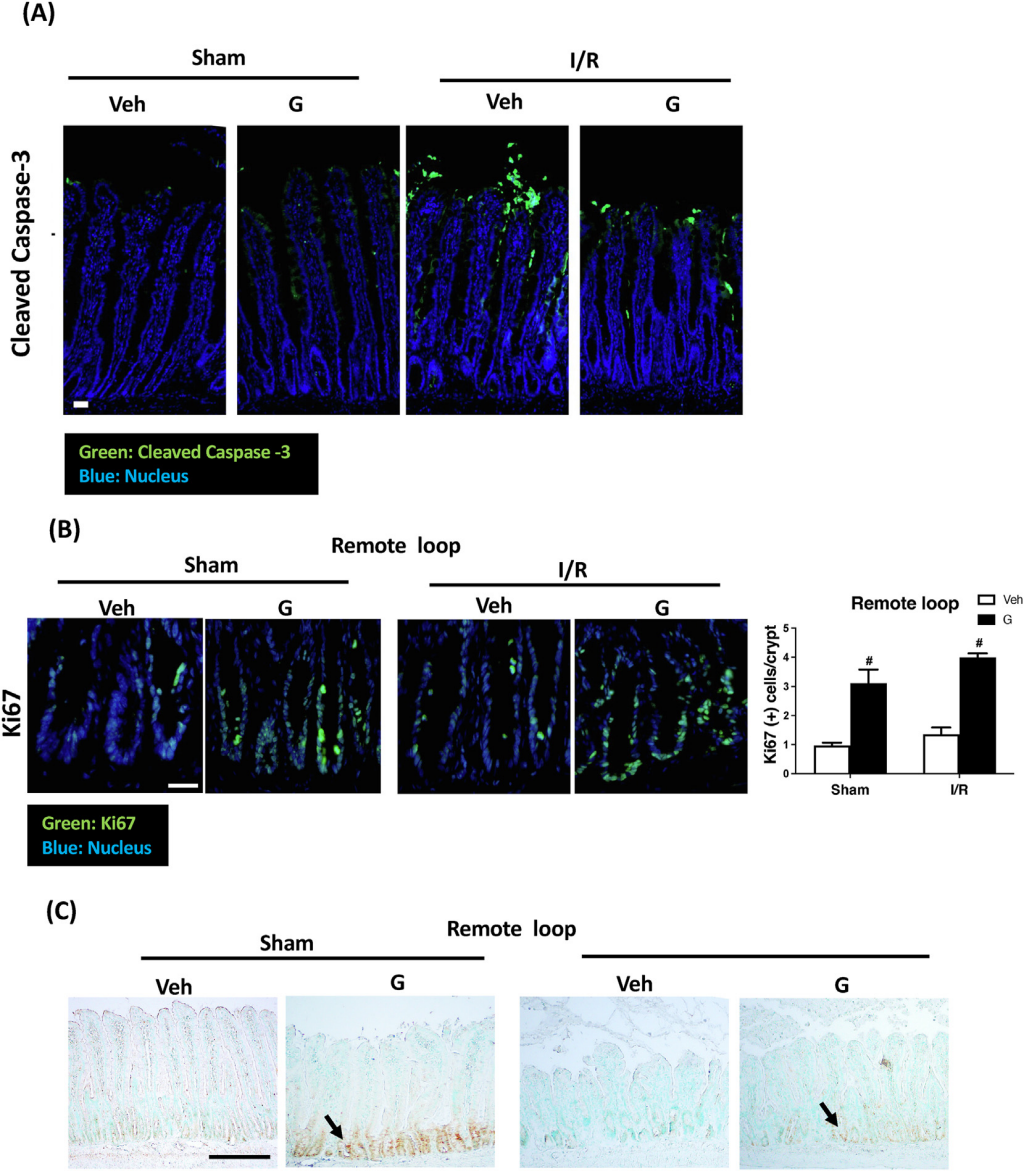
Local enteral glucose reduces I/R-induced caspase-3 cleavage and restores crypt activity in remote intestine. (A) I/R increases the number of cleaved caspase-3-positive cells (green) in the remote loop. This effect was markedly reduced in rats receiving local glucose treatment (indicated as G) (Scale bar: 20 μm). **(**B) Increased Ki67 (green)- and (C) PCNA-positive cells were seen in remote intestinal loops of rats treated with local enteral glucose (Scale bar: 50 μm for B; 100 μm for C). The fluorescence intensities of Ki67-positive cells were quantified from 5 well-oriented crypts with a longitudinal view for each animal; a total of 6 rats per group were analyzed. (*N* = 6–8/group, ∗*P* < 0.05 vs. Sham + Veh, ^#^*P* < 0.05 vs. IR + Veh). G, glucose; I/R, ischemia/reperfusion; PCNA, proliferating cell nuclear antigen; Veh, vehicle.

We next wanted to evaluate whether the local glucose treatment can modulate I/R-induced cell death in intestinal tissues. As expected, I/R caused significant cell apoptosis, as evidenced by an abundance of cleaved caspase-3-positive cells at the villus tips in both the local and remote loops. However, enteral administration of glucose to the local loop had an inhibitory effect on cleaved caspase-3 accumulation in both the local and remote villus epithelial cells (Figure 3A, Supplemental Figure 2A). Local and remote ischemic tissues also displayed decreased levels of epithelial proliferation, as indicated by reductions in Ki67 and PCNA immunoreactivities in the crypt regions. However, luminal administration of glucose in the local jejunum increased crypt Ki67 and PCNA staining in the remote and local ischemic intestine (Figure 3B, C, Supplemental Figure 2B, C). Quantification of the results showed that I/R significantly reduced the number of Ki67-positive cells per crypt, and this phenomenon was reversed in both the local and remote loops by treatment of glucose in the local jejunum (Figure 3B, Supplemental Figure 2B).

### Local enteral glucose administration prevents loss of goblet cells and mucus secretion in the remote intestine

**FIGURE 4:**
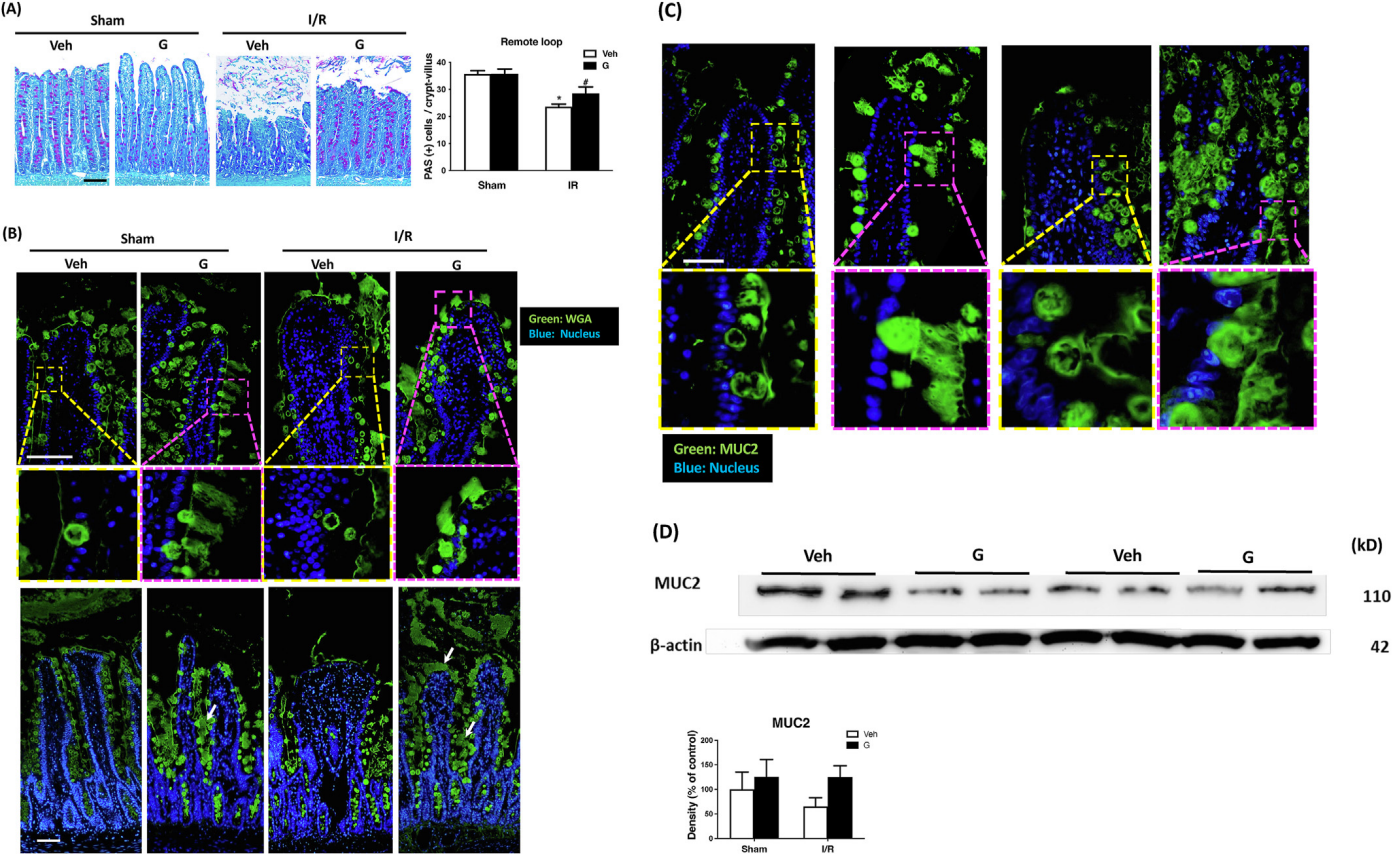
Administration of glucose in the local intestinal lumen restores remote intestinal goblet cell numbers and enhances mucus secretion in I/R-challenged tissues. (A) Goblet cells were labeled with Periodic Acid-Schiff (PAS) and appeared with a purple/red color in the images. A reduction in goblet cell numbers was observed in the remote intestine of I/R-treated rats compared with Sham controls. Administering glucose (indicated as G) to the local loop counteracts this decrease. Five random complete villus-crypt images were selected for goblet cell quantification for each animal; a total of 6 rats per group were analyzed (*N* = 6–8/group, ^∗^*P* < 0.05 vs. Sham + Veh, ^#^*P* < 0.05 vs. I/R + Veh.) Scale bar: 100 μm. **(**B) Wheat Germ Agglutinin (WGA, green) and (C) MUC2 (green) staining of mucus and Hoechst staining of nuclei (blue) in the remote intestine. In the absence of glucose treatment to the local loop, mucus within goblet cells exhibits cavitation (yellow box) in both Sham and I/R-treated rats. After enteral glucose administration in the local loop, mucus fills the goblet cells and displays a jet-like secretion pattern (pink box). The spaces between the villi are also filled with mucus (white arrow). (*N* = 6–8/group, Scale bar: 50 μm). (D) Western blotting for MUC2 in remote intestinal mucosa scrapings shows no significant differences between the groups. G indicates glucose treatment. (*N* = 6–8/group). G, glucose; I/R, ischemia/reperfusion; Veh, vehicle.

We further investigated the protective effects of local glucose on the remote intestine after I/R by conducting PAS staining for goblet cells. We found that I/R led to a significant decrease in the number of goblet cells in both local (Supplemental Figure 3) and remote (Figure 4A) intestinal loops. However, administration of glucose in the local jejunum significantly increased in the numbers of goblet cells present in both the local and remote jejunum (Supplemental Figure 3 and Figure 4A).

Next, we utilized WGA and MUC2 antibody to label mucus in the remote jejunum. To perform mucus staining on intestinal segments, it is necessary to ligate both ends of the segment during sample collection to prevent the loss of water-soluble mucus during tissue washing. In the local loop, the initial injection of Krebs buffer with or without glucose can cause water-soluble mucus loss. Therefore, in the local loop, we only performed goblet cell staining and did not conduct mucus staining. The mucus staining was only performed on the remote loop. In the absence of glucose treatment, sham-operated and I/R rats exhibited signs of vacuolization and loss of mucus (Figure 4B) and MUC2 protein (Figure 4C) in the goblet cells. However, after administering glucose in the local jejunum, goblet cells in the remote intestine became filled with mucus and even showed signs of jet-like secretion. The secreted mucus covered the epithelial cell surfaces and filled the spaces between the villi (Figure 4B, C). The interstices between the villi are similarly saturated with mucus after local glucose administration (Figure 4B). Western blotting further revealed a trend toward decreased expression of MUC2 protein in the remote jejunum of the I/R group compared with sham-operated tissues, and MUC2 levels also showed a trend toward increase after glucose administration. However, the differences between groups did not reach statistical significance (Figure 4D).

### I/R-triggered total BT and enteric mucosal inflammation in the remote intestine are alleviated by local enteral administration of glucose

**FIGURE 5:**
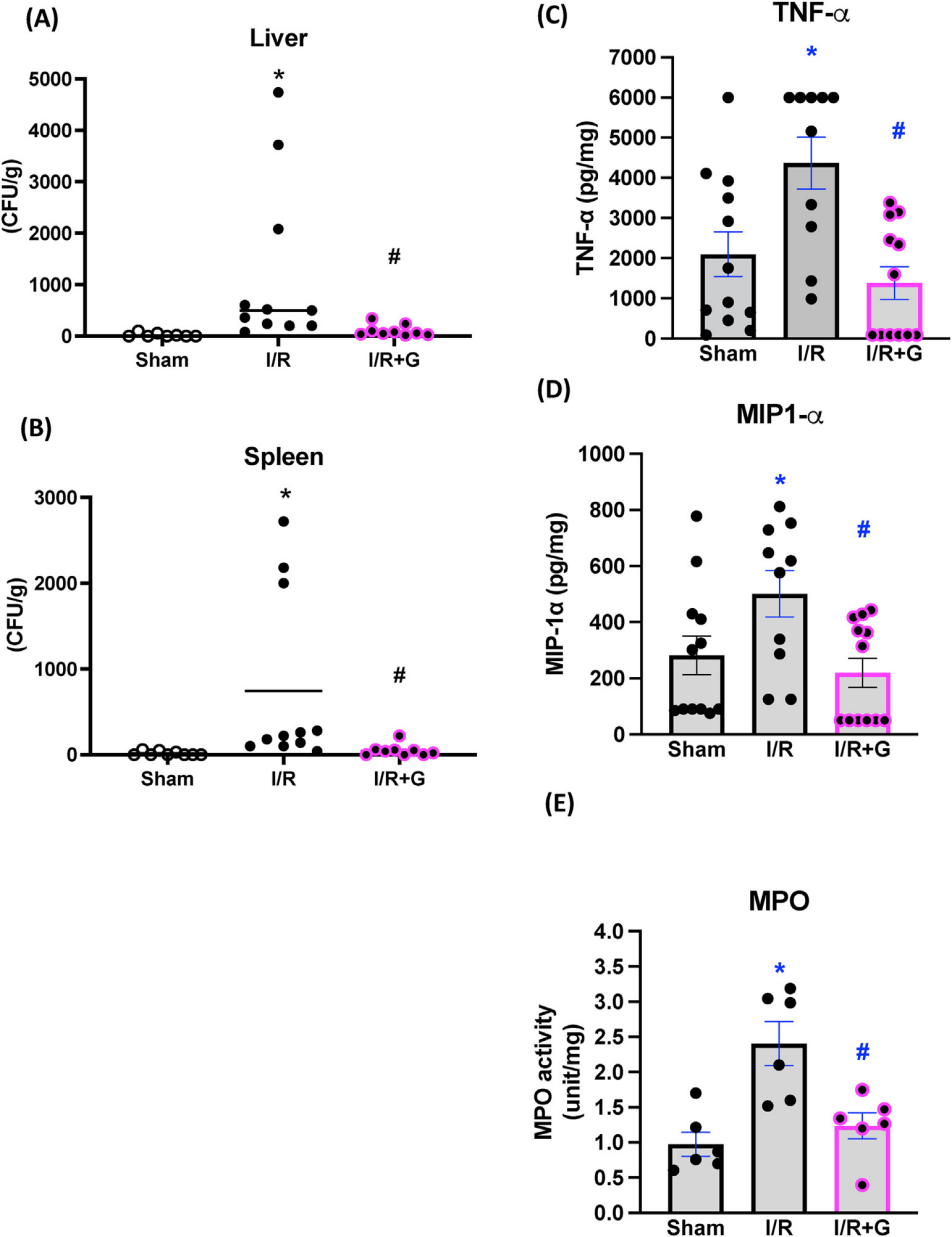
Local enteral glucose treatment prevents I/R-induced total bacterial translocation and mucosal inflammation in the remote intestinal loop. The bacterial CFU was calculated and normalized to per gram of liver (A) and spleen (B) tissues. (*N* = 10–12/group, ^∗^*P* < 0.05 vs. Sham, ^#^*P* < 0.05 vs. IR.). The jejunal tissue of sham, I/R and I/R + glucose (indicated as G) groups were processed. Measurements were made of (C) myeloperoxidase (MPO) activity, (D) tumor necrosis factor (TNF)-α level, and (E) macrophage inflammatory protein (MIP)-1α level. Local enteral glucose decreased the levels of MPO activity and mucosal proinflammatory cytokine production in the remote loop. (*N* = 10–12/group, ^∗^*P* < 0.05 vs. Sham, #*P* < 0.05 vs. IR). CFU, colony-forming unit; I/R, ischemia/reperfusion; MPO, myeloperoxidase.

Consistent with our previous research [13], local administration of glucose reduced overall BT into liver and spleen (Figure 5A, B). Based on our observations up to this point, we expected that the protective effects of glucose on remote intestinal segments were probably mediated by enhanced mucus secretion that prevented contact between intestinal bacteria and the mucosa. Such an effect would be reflected by a lack of mucosal inflammation. Therefore, we predicted that glucose-induced mucus secretion should prevent mucosal inflammation, and we assessed the state of mucosal tissue inflammation. I/R triggered an increase in intestinal MPO activity (Figure 5C) and elevated levels of mucosal TNF-α and MIP-1α (Figure 5D, E). However, the administration of local luminal glucose significantly reduced I/R-induced mucosal inflammation, as assessed by MPO activity and levels of TNF-α and MIP-1α (Figure 5C–E). These findings suggest that glucose-stimulated mucus secretion may reduce bacterial attachment and infiltration to mitigate I/R-associated mucosal inflammation.

## Discussion

In this study, we build upon our previous findings that luminal administration of glucose or glycolytic metabolites in the jejunal loop protects against I/R-induced epithelial death and intestinal injury through PI3K/Akt and receptor-interacting protein (RIP) signaling [12,13]. Here, we discovered that local enteral glucose could stimulate goblet cell-mediated mucus secretion to protect I/R-induced remote barrier dysfunction and BT. Damage to the mucosal tissue, loss of tight junctional proteins, permeability increases, apoptosis, and decreased crypt activity were induced by I/R in vehicle-treated rats but could be attenuated by local glucose administration. Additionally, in the absence of glucose in the local loop, reductions in goblet cell numbers and mucus secretion were accompanied by cellular vacuolization. When glucose was administered, the number of goblet cells was increased, and the cells were filled with and secreted mucus that covered the spaces between villi. As mucus loss facilitates bacterial adhesion and invasion to cause inflammation, we found that stimulation of mucus secretion in the remote loop corresponded to a reduced inflammatory response in the region. This finding may help to explain our previous findings that administering glucose in the jejunal loop prevented global BT to the liver and spleen. Our previous study showed that glycolytic metabolites, pyruvate and ATP can all suppress epithelial cell death and improve crypt function in the local jejunum [12]. This study further shows that local glucose treatment can protect barrier function and enhance the functionality of goblet cells in local and remote intestinal tissues to protect against I/R injury.

MUC2 is the predominant component of the intestinal mucus layer function as its structural skeleton. In the ER of the goblet cell, MUC2 is present as a dimer, and its central mucin domains are flanked by an N-terminal part and a C-terminal portion. In the Golgi, the dimerized MUC2 is rearranged into a trimer through intermolecular disulfide bridges. Importantly, the transfer of MUC2 oligomers from ER to the cis side of the Golgi necessitates the presence of ATP [26,27]. In our study, glucose-mediating ATP production may assist the intercellular transportation of MUC2. Then, the MUC2 is packaged densely into secretory vesicles as mucin oligomers. The low pH, high calcium, and low water content of secretory vesicles are key factors promoting oligomeric mucin storage. Secretion of the vesicles is then triggered by hydrogen carbonate (HCO_3_^−^) secretion by cystic fibrosis transmembrane conductance regulator channel in surrounding enterocytes. The secretion of HCO_3_^−^ generates alkalizing conditions and acts together with calcium chelation to lower the MUC2 concentration compared with that in the mucus granules. These microenvironmental changes cause vast expansion of the mucus oligomers, unfolding each highly organized 2D sheet like an umbrella, which interacts with previously formed sheets to build a 3-dimensional mucus layer that can expand >1000-fold in volume [28,29]. Typically, the normal mucus barrier supports a plentiful and varied population of microorganisms in the gut while also protecting the epithelium and immune system from direct contact with bacteria, thereby preventing unnecessary immune responses [4]. Most studies on the role of mucus in intestinal barrier function have focused on colonic diseases. Early research has found that MUC2 deficiency leads to colonic inflammation in mice, which spontaneously develops colitis [30] and later on colorectal cancer [31]. In patients with ulcerative colitis, the reduction in mucus structural components allows bacterial penetration to the inner mucus layer and contact with the epithelium, which serves as a cause and an early event in the pathogenesis [32,33]. Recent studies have also found that dietary factors, such as a Western-style diet or dietary emulsifiers, can adversely affect the mucus layer, allowing pathogenic bacteria to encroach into the mucus of both the large and small intestines [34].

Our study demonstrated that glucose can simultaneously increase the number of goblet cells and enhance MUC2 mucus secretion during the I/R challenge. In the intestine, goblet cells originate from secretory lineage cells derived from crypt stem cells. Low levels of Notch signaling promote goblet cell differentiation [15]. The glucose-mediated PI3K/Akt and glycolytic ATP may play a role in promoting epithelial renewal following ischemic stress [12,13], which may contribute to the regeneration of goblet cells. An early study found that the administration of a glucose-containing total parenteral nutrition (TPN) solution in rats led to the depletion of mucus and an elevation of permeability in the small intestine [35]. Unlike the results from glucose-containing TPN solution, our current study emphasized the importance of enteral administration of glucose by reinforcing the mucus barrier and lowering intestinal permeability. The precise mechanism by which luminal glucose stimulates mucus secretion remains unknown. One possible mechanism in the regulation of mucus secretion is through the reactive oxygen species (ROS) produced by intestinal NADPH oxidases (NOX) and dual oxidases (DUOX) [36]. A study revealed that the absence of autophagy proteins and NADPH oxidase leads to disruptions in the accumulation of mucin granules in colonic goblet cells. NOX generates ROS in goblet cells, which increases mucus release in colonic goblet cells [37]. Furthermore, a newly identified “sentinel” goblet cell (senGC), localized at the entrance of the colonic crypt, activates the TLR downstream Nlrp6 inflammasome, triggering TLR- and MyD88-dependent NOX/DUOX ROS synthesis, which in turn stimulates the exocytosis of Muc2 mucin from the senGC [38]. Our unpublished data showed that glucose could increase NOX1 subunit expression in the crypt region after I/R. However, research on the detailed mechanisms between enteral glucose and goblet cells is still lacking and requires further investigation.

Although we did not investigate the specific molecular mechanisms by which local glucose confers protection to the remote intestine in this study, distal regulation of intestinal tissues is known to occur via many pathways. Hormones, neural signals, and immune cells may all participate in such regulatory processes. The gastrointestinal tract is the largest endocrine organ and has extensive chemosensing functions. For instance, L-type enteroendocrine cells (L cells) are equipped with heterodimeric sweet taste T1R2/T1R3 (taste 2 receptor/taste 3 receptor) metabotropic G protein-coupled receptors (GPCRs) that are activated by oral administration of glucose and trigger downstream secretion of glucagon-like peptide (GLP)-1 and GLP-2 [39]. Notably, activation of T1R2/T1R3 GPCRs on L cells stimulates the release of GLP-1, which affects glucose homeostasis and upregulates SGLT1 expression in adjacent enterocytes. Moreover, L cells have been identified in the jejunum and distal gut [39,40]. GLP-2 is a 33-residue peptide with tissue-protective and -regenerative functions; it is derived from proteolytic cleavage of proglucagon by prohormone convertase 1/3. In addition to its upregulation by glucose, intestinal GLP-2 plasma levels rise rapidly after intestinal injury or major intestinal resection. Thus, GLP-2 may have the capacity to integrate glucose and gut injury signals [41]. Of note, PI3K is required for GLP-2-mediated cellular protection [42,43]. Nevertheless, further research is needed to determine if GLP-2 is involved in the remote protective effects of glucose and mediates increases in goblet cell numbers and mucus secretion.

A standard treatment for acute diarrhea is the administration of an oral rehydration solution, which consists mainly of sodium chloride, potassium chloride, bicarbonate, and glucose [44,45]. Absorption of glucose occurs via SGLT-1 in small intestine epithelial cells, and this action not only prevents damage to these cells but also maintains their barrier function [44,[46], [47], [48]]. *In vitro* studies using human intestinal epithelial Caco-2 cells have shown that glucose uptake via SGLT1 inhibits apoptosis and prevents barrier disruption caused by bacterial LPS [49]. In an *in vitro* model of inflammatory bowel disease, low glucose condition induced hyperpermeability via downregulation of tight junctional molecules [50]. In addition to its modulation of tight junctional proteins, glucose also has a substantial impact on the immunological barrier. A delicate study also demonstrates that stromal cells derived from lymph nodes rely on glycolysis to produce CXCL13 and maintain a functional immune barrier [51]. In a murine model of septic shock, oral administration of glucose was found to activate SGLT1, resulting in 100% protection of mice from lethal endotoxic shock induced by intraperitoneal LPS administration. This protective effect is attributed to an increase in the anti-inflammatory cytokine IL-10 [52]. Our previous and current studies showed that administration of glucose in the intestinal lumen during I/R can prevent BT, protect small intestinal epithelial cells from cell death, and enhance mucus secretion by jejunal goblet cells. These findings highlight the importance of timing and dosing of glucose administration for maintaining normal intestinal function [12,13,53]. Although oral rehydration therapy has become standard for the clinical treatment of acute diarrhea, there are still no clearly defined treatment guidelines for conditions involving SMA I/R [9]. In the current study, our findings indicate a plausible direction for future clinical management of small intestinal I/R, especially under predictable scenarios.

Previous research has shown that the colon is relatively resistant to I/R injury compared with the small intestine [21,22]. One possible reason for this difference in susceptibility is the presence of a dual-layer mucus system in the colon. Following colonic I/R, the colon tissue can reload its goblet cells and continuously secrete MUC2, which helps prevent bacterial invasion and maintain colonic barrier function. Previous studies have also revealed that ischemia in the right colon is more likely to lead to severe disease than ischemia on the left side, as the right colon has a less dense inner mucus layer [3,21,23]. In contrast, the small intestine has only a single mucus layer. Under normal physiologic conditions, the small intestine has a lower bacterial load than the colon and does not need to continuously produce mucus to protect from its resident bacterial populations. However, we found that during I/R, administration of luminal glucose can help maintain the physical mucus barrier in the small intestine, which can prevent BT and mucosal inflammation to preserve the overall intestinal barrier function. As such, our study suggests a promising therapeutic strategy for future clinical applications.

In conclusion, our data show that mesenteric I/R leads to impaired barrier function and loss of the mucus layer from the epithelial villi in the small intestine. This loss of mucus may result in the enterocytic epithelium being directly exposed to the intraluminal contents. The intraluminal bacteria may then penetrate and translocate through the intestine during I/R challenge. By administering enteral glucose, we were able to preserve both local and remote intestinal barrier functions. Additionally, treatment-induced increases in crypt activity and goblet cell numbers maintained the mucus barrier and prevented mucosal inflammation. Thus, our results suggest that the glucose-triggered production of mucus by goblet cells plays a crucial role in protecting the small intestine from BT and inflammation caused by bacterial contacting and maintains the integrity of the intestinal barrier after I/R. Our results provide a more precise barrier protection role of enteral glucose treatment upon I/R challenge, presenting new opportunities and evidence for future therapeutic potential in critical care nutrition.

## Author contributions

The authors’ responsibilities were as follows – C-YH: conceptualization, supervision, funding acquisition, writing – review and editing; C-YH, T-YG, W-TK, Y-ST: methodology; T-YG, W-TK: software; C-YH, T-YG: validation investigation, data curation, and writing – original draft preparation; C-YH, T-YG, W-TK: formal analysis; C-YH, LC-HY: resources; and all authors: read and approved the final manuscript.

## Conflict of interest

The authors report no conflicts of interest.

## Funding

This research was funded by National Science and Technology Council (109-2320-B-005-007, 111-2320-B-005-002).

## Data availability

Data described in the manuscript will be made available upon request pending application and approval.

## Ethics approval

All animal experiments were approved by the Institutional Animal Care and Use Committee (IACUC) of National Chung Hsing University. IACUC No.: 108-184 & 110-126.
