# Supplementary material for: Glucose-Stimulated Mucus Secretion by Goblet Cells Mitigates Intestinal Barrier Dysfunction in a Rat Model of Mesenteric Ischemia/Reperfusion Injury
Source: Curr Dev Nutr. 2024 Jul 26;8(9):104431. doi: 10.1016/j.cdnut.2024.104431 (PMC11388543; doi:10.1016/j.cdnut.2024.104431)

Article Title: Glucose-stimulated mucus secretion by goblet cells mitigates intestinal barrier dysfunction in a rat model of mesenteric ischemia/reperfusion injury

Ting-You Guo et al.

Supplementary figure legends

Supplementary Figure 1. Enteral glucose treatment ameliorated mesenteric ischemia/reperfusion (I/R)-induced barrier defects in the small intestine. Intestinal tissue sections from local small jejunal loops were processed for H&E staining. **(A)** Representative histological images are shown. I/R-induced mucosal destruction was significantly ameliorated by enteral glucose treatment (indicated as G) (Scale bar: 100 μm). Photomicrographs of small intestine tissues stained for tight junctional proteins **(B)** ZO-1 (green) and **(C)** occludin (green). Nuclei were stained with Hoechst (blue). Arrows indicate sites of apical surface disruption in ZO-1- and occludin-stained intestines after I/R. The disruption of tight junction proteins was prevented by local enteral glucose treatment. **(D)** Goblet cells were labeled with Periodic Acid-Schiff (PAS) and appear with purple/red color in the images. The reduction in goblet cell numbers observed in I/R-exposed jejunum was reversed by enteral administration of glucose. Five random complete villus-crypt images were selected for goblet cell counting in each group. (N = 6-8/group, * $p < 0.05$ vs. Sham + Veh, # $p < 0.05$ vs. IR + Veh; **B-C** Scale bar: 20 μm).

Supplementary Figure 2. Enteral glucose reduces I/R-induced caspase 3 cleavage and restores crypt activity. (A) I/R-induced increases in the number of cleaved caspase-3-positive cells (green) in the jejunum were abrogated in the enteral glucose-treated (indicated as G) rats. (Scale bar: 20 μm) **(B)** Increased Ki67- and PCNA-positive cell numbers were observed in jejunal tissues after enteral glucose administration. The fluorescence intensities of Ki67-positive cells were quantified from 9 well-oriented crypts in longitudinal view from each animal; a total of six rats per group were analyzed. (N = 6-8/group, * $p < 0.05$ vs. Sham + Veh, # $p < 0.05$ vs. IR + Veh.) (Scale bar: 50 μm for **B**; 100 μm for **C**).

Supplementary Figure 3. Administration of glucose in the local intestinal lumen restores the number of jejunal goblet cells after I/R challenge. Goblet cells were

labeled with Periodic Acid-Schiff (PAS) and appear with purple/red color in the images. A reduction in goblet cell numbers was observed in the local intestine of I/R-exposed rats, as compared to Sham controls. Enteral glucose (indicated as G) counteracted the decrease in goblet cell numbers. Five random complete villus-crypt images were selected for goblet cell quantification for each animal; a total of six rats per group were analyzed. (N = 6-8/group, * $p < 0.05$ vs. Sham + Veh, # $p < 0.05$ vs. I/R + Veh. Scale bar: 100 μm).

Supplementary Figure 1

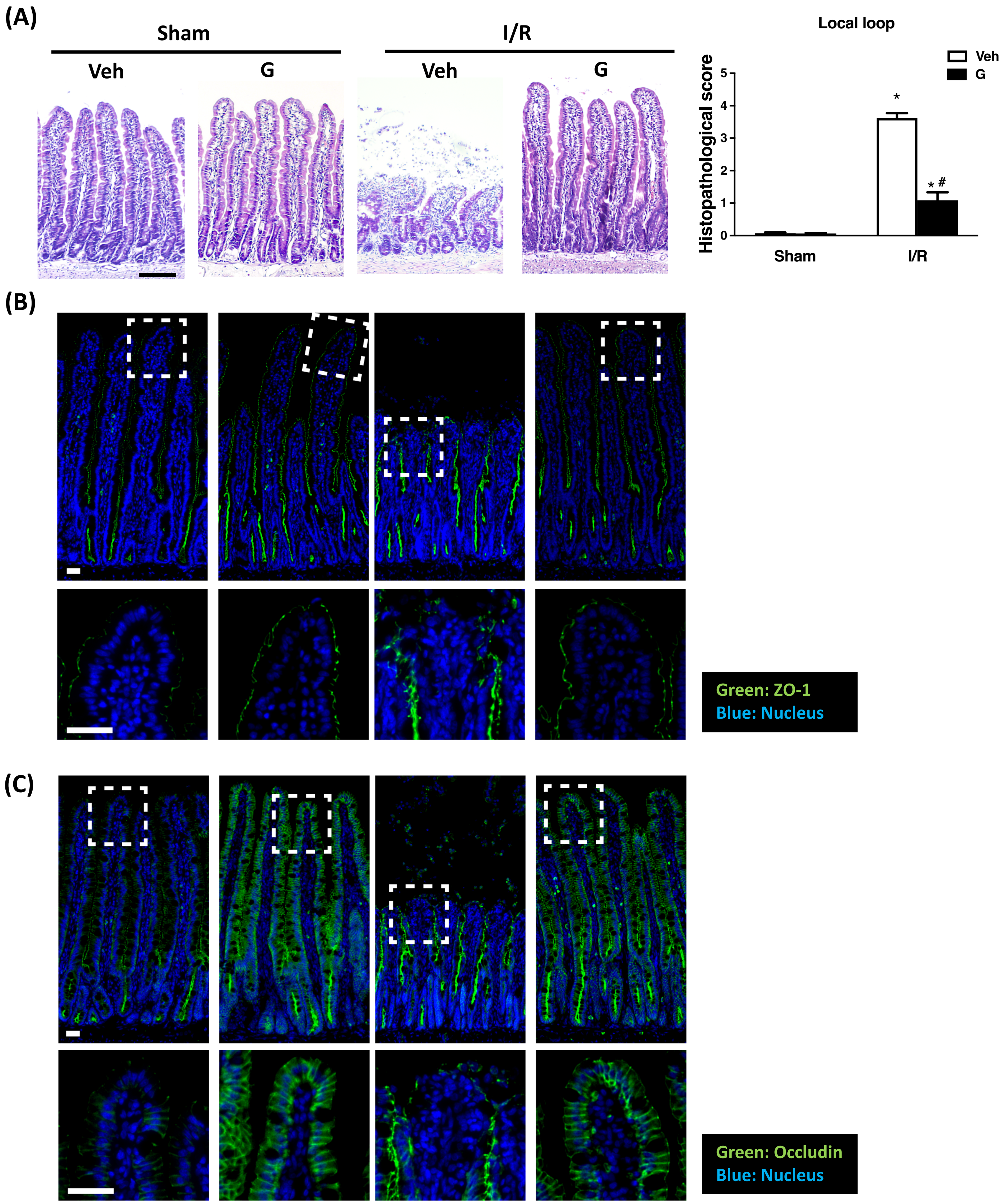

Supplementary Figure 2

(A)

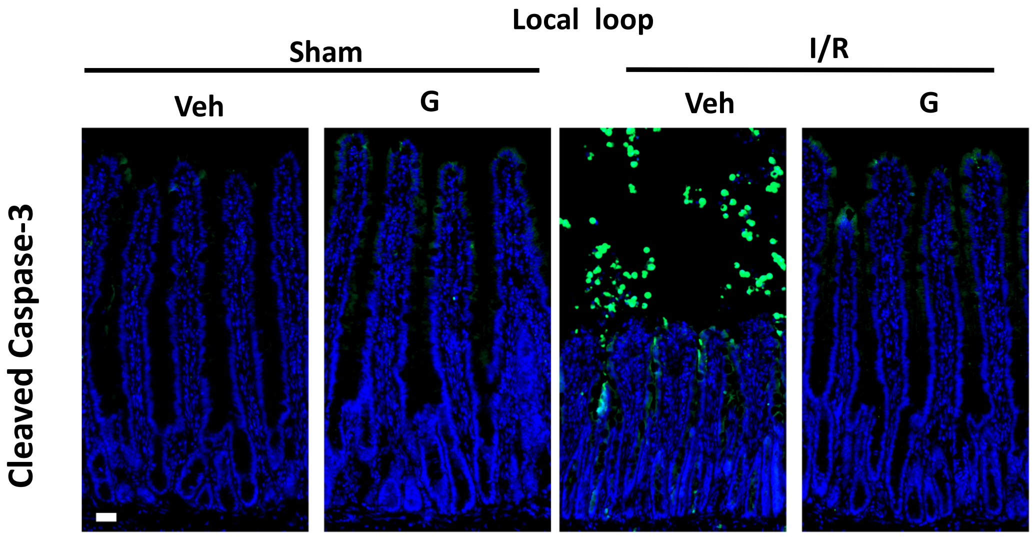

Green: Cleaved Caspase -3
Blue: Nucleus

(B)

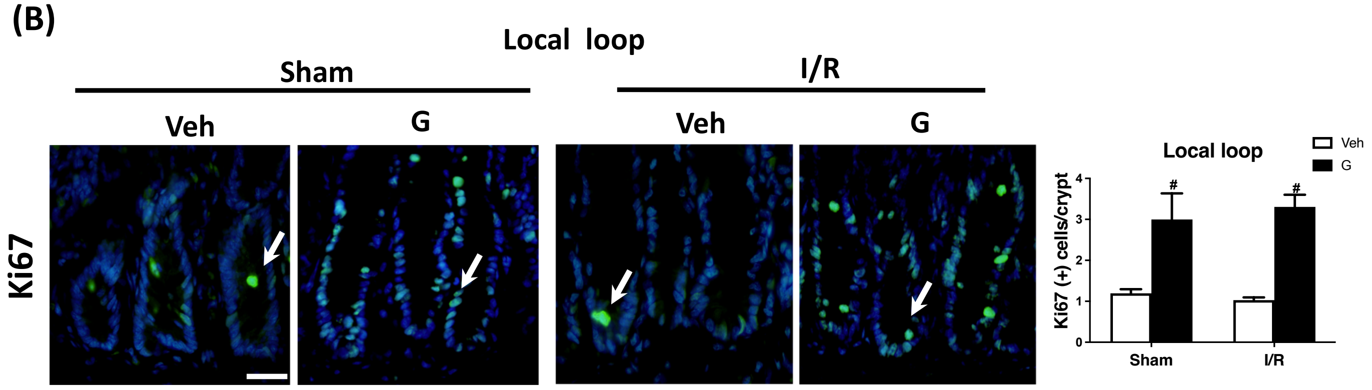

Green: Ki67
Blue: Nucleus

(C)

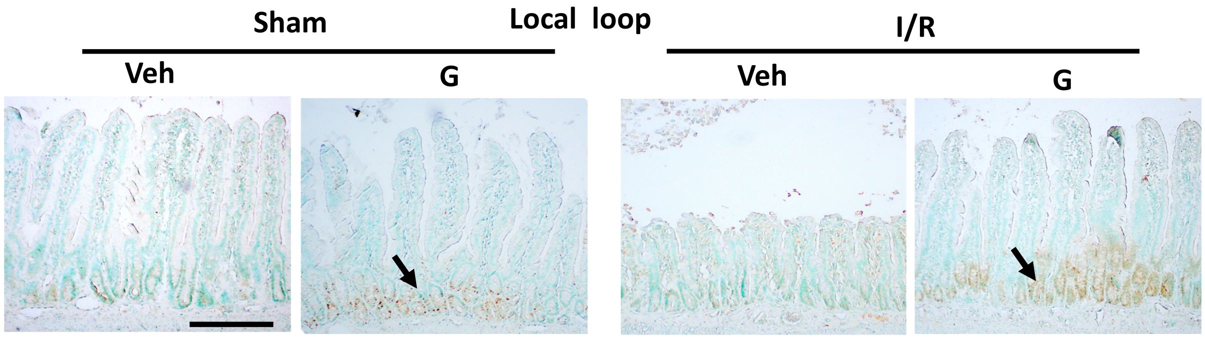

Supplementary Figure 3

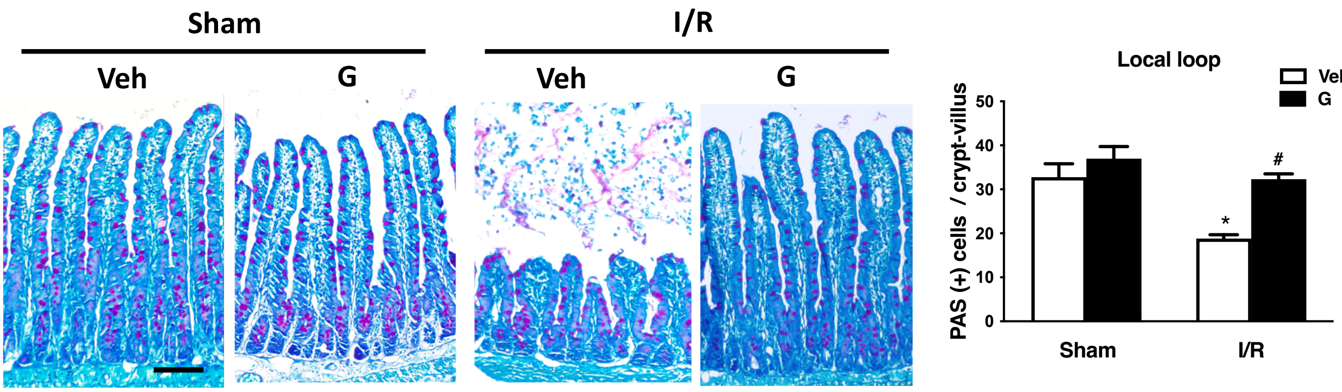

Supplement: multimedia component 1 [file mmc1.pdf]
